# The Carboxy-Terminal Domain of *Dictyostelium* C-Module-Binding Factor Is an Independent Gene Regulatory Entity

**DOI:** 10.1371/journal.pone.0005012

**Published:** 2009-04-03

**Authors:** Jörg Lucas, Annika Bilzer, Lorna Moll, Ilse Zündorf, Theodor Dingermann, Ludwig Eichinger, Oliver Siol, Thomas Winckler

**Affiliations:** 1 School of Biology and Pharmacy, Institute of Pharmacy, Department of Pharmaceutical Biology, University of Jena, Jena, Germany; 2 Institute for Biochemistry I, Medical Faculty, University of Cologne, Cologne, Germany; 3 Institute of Pharmaceutical Biology, University of Frankfurt, Frankfurt am Main, Germany; Brunel University, United Kingdom

## Abstract

The C-module-binding factor (CbfA) is a multidomain protein that belongs to the family of jumonji-type (JmjC) transcription regulators. In the social amoeba *Dictyostelium discoideum*, CbfA regulates gene expression during the unicellular growth phase and multicellular development. CbfA and a related *D. discoideum* CbfA-like protein, CbfB, share a paralogous domain arrangement that includes the JmjC domain, presumably a chromatin-remodeling activity, and two zinc finger-like (ZF) motifs. On the other hand, the CbfA and CbfB proteins have completely different carboxy-terminal domains, suggesting that the plasticity of such domains may have contributed to the adaptation of the CbfA-like transcription factors to the rapid genome evolution in the dictyostelid clade. To support this hypothesis we performed DNA microarray and real-time RT-PCR measurements and found that CbfA regulates at least 160 genes during the vegetative growth of *D. discoideum* cells. Functional annotation of these genes revealed that CbfA predominantly controls the expression of gene products involved in housekeeping functions, such as carbohydrate, purine nucleoside/nucleotide, and amino acid metabolism. The CbfA protein displays two different mechanisms of gene regulation. The expression of one set of CbfA-dependent genes requires at least the JmjC/ZF domain of the CbfA protein and thus may depend on chromatin modulation. Regulation of the larger group of genes, however, does not depend on the entire CbfA protein and requires only the carboxy-terminal domain of CbfA (CbfA-CTD). An AT-hook motif located in CbfA-CTD, which is known to mediate DNA binding to A+T-rich sequences *in vitro*, contributed to CbfA-CTD-dependent gene regulatory functions *in vivo*.

## Introduction

*Dictyostelium discoideum* is a unicellular, amoeboid organism that lives in the soil and feeds on bacteria. When the environmental conditions limit vegetative growth, amoebae collect into multicellular aggregates in which the cells differentiate into stalk and spore cells of the future fruiting bodies [1]. Cyclic AMP (cAMP) coordinates both the aggregation and multicellular development of *D. discoideum*; it acts as a chemoattractant and as a morphogen. Aggregation-competent *D. discoideum* cells sense cAMP by means of the cAMP-specific, G protein-coupled receptors CAR1–3 [2]. Signaling by CAR1 during aggregation leads to the activation of effector enzymes such as adenylyl cyclase (ACA) and extracellular signal-related kinase 2 (ERK2) by both G protein-dependent and -independent mechanisms. Protein kinase A, activated by elevated intracellular cAMP levels, phosphorylates downstream substrates and mediates the induction of genes required for aggregation and post-aggregation development. Since the gene products required for the production and sensing of cAMP are themselves induced by cAMP, a positive feedback loop is established that is required for the full induction of genes that regulate multicellular development [2].

Although cAMP-dependent induction of multicellularity in *D. discoideum* has been studied intensively both biochemically and genetically, little is known about the transcription factors that support gene expression at the transition from growth to development. For example, it has been suggested that Myb2 is a transcription factor involved in the induction of ACA, since *mybB* null cells have an aggregation phenotype similar to that of *acaA* null cells [3], [4]. Interestingly, a similar phenotype is observed when cells are depleted of CbfA, the C-module-binding factor [5]. CbfA was first described in experiments unrelated to multicellular development. The factor was isolated biochemically from growing cells, guided by its specific binding to a putative transcription regulatory element, the C-module, within the *D. discoideum* retrotransposon TRE5-A [6], [7]. Later, the *cbfA* gene was cloned [7] and a mutant was created that underexpressed CbfA due to the partial suppression of a premature *amber* stop codon by a co-expressed *amber* suppressor tRNA gene [8]. The *cbfA^am^* mutant JH.D stably underexpresses CbfA at about 5% of the wildtype level and shows a strong aggregation defect with an overall developmental phenotype similar to *acaA* null cells [5], [8]. We recently showed that CbfA binds to the *acaA* upstream region *in vitro*, and that CbfA is required to induce *acaA* promoter activity during aggregation, suggesting that CbfA is an essential regulator of ACA expression [5], [9].

CbfA is a multidomain protein that belongs to the large family of “jumonji-type transcription regulators”, which contribute to the deciphering of the histone code by removing methyl groups from methyllysine or methylarginine residues in histone tails [10], [11]. A “carboxy-terminal jumonji domain” (JmjC), named after the murine jumonji protein [12], catalyzes this type of oxidative demethylation reaction (reviewed in [10]). A JmjC domain is located at the amino terminus of the CbfA protein (amino acids 113–280). CbfA further contains two zinc finger-like regions located at positions 373–414 and 492–550 of the protein, followed by a distinct region of 217 amino acids length that consists of 50% asparagine residues. This asparagine-rich domain separates the zinc fingers from a carboxy-terminal domain (CTD) that spans 230 amino acids and contains a peptide motif similar to class III AT-hooks [13]. An AT-hook is a small DNA-binding motif defined by a glycine-arginine-proline (GRP) tripeptide in which the central arginine is essential for DNA minor groove binding (DKPKGRPPKNLKEW in CbfA; underlined residues identical to the most frequent amino acids in these positions in other AT-hook proteins). AT-hooks were first discovered in members of the high mobility group of non-histone (HMG) chromatin proteins [14], [15], in which they mediate, via DNA binding, the assembly of nucleoprotein-DNA transcriptional complexes [16], [17]. AT-hook motifs are also found in other non-HMG proteins in which they appear to be necessary elements for the cooperation of DNA-binding activities in the context of transcription complexes [13]. The AT-hook of CbfA is sufficient to mediate the binding of CbfA-CTD to the C-module of retrotransposon TRE5-A *in vitro*, with a specificity similar to the full-length CbfA protein [7].

One aspect of the phenotype of CbfA-depleted mutants such as strain JH.D [5] is that they show reduced steady-state levels of transcripts derived from retrotransposon TRE5-A (O.S. and T.W., manuscript in preparation). We observed that when either full-length CbfA or the CbfA-CTD were expressed in the *cbfA^am^* background of JH.D cells, transcript levels of TRE5-A were fully restored, whereas multicellular development was rescued by full-length CbfA but not by CbfA-CTD (O.S. and T.W., manuscript in preparation). Thus, we speculate that CbfA-CTD displays gene regulatory functions that do not depend on the remainder of the CbfA protein, particularly the JmjC domain that is thought to function through defined chromatin modifications. This hypothesis is supported by results from this study, in which we performed genome-wide expression profiling of vegetatively growing wildtype *D. discoideum* cells in comparison with the *cbfA^am^* mutant JH.D. We found that full-length CbfA has both transcription activating and repressing activities, and that the factor regulates more than 160 genes of the *D. discoideum* genome. We compared these data with both strains expressing CbfA-CTD and show that the majority of CbfA-dependent genes are regulated by CbfA-CTD alone.

## Results

### Genome-wide evaluation of CbfA-dependent gene expression

We performed cDNA microarray analyses to evaluate the global gene regulatory functions of CbfA in vegetatively growing *D. discoideum* amoebae. The cDNA microarrays used in this study carried a non-redundant set of 5,423 expressed sequence tag clones and, in addition, sequences of 450 selected genes, which were all spotted in duplicate [18]. Thus, the DNA microarrays represented about half of the estimated 12,500 genes of the *D. discoideum* genome [19]. We performed comparative DNA microarray hybridizations with three independently prepared RNA samples of wildtype strain AX2 and the CbfA-depleted mutant JH.D and used them on six microarray slides. Differentially expressed genes were determined by combining the data from six microarrays, followed by statistical analysis [20]. Without threshold, a total of 473 individual genes were found to be aberrantly expressed in JH.D cells. Of these genes, 238 were upregulated and 235 were downregulated in JH.D cells. Applying a threshold of >1.5-fold change reduced the number of genes reported as differentially expressed to 162 (82 upregulated in JH.D cells, 80 downregulated). The complete set of 162 CbfA-regulated genes identified in this study is listed in Table S1. Only these genes were considered in subsequent analyses.

**Figure 1:**
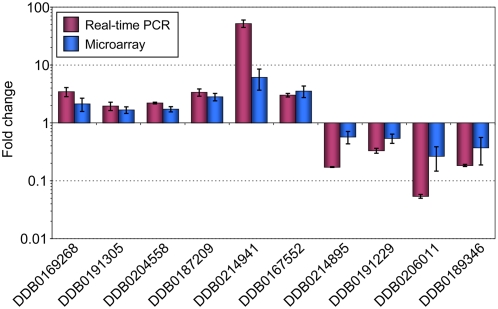
Validation of DNA microarray results by real-time RT-PCR. Ten representative ESTs from the microarray data, indicated by the corresponding Dictybase (DDB) numbers, were analyzed by real-time RT-PCR. Note that expression of genes is calculated for AX2 vs. JH.D, which means that expression AX2>JH.D results in ratios>1. The data are expressed as means of fold change±SD from three independent RNA preparations.

**Figure 2:**
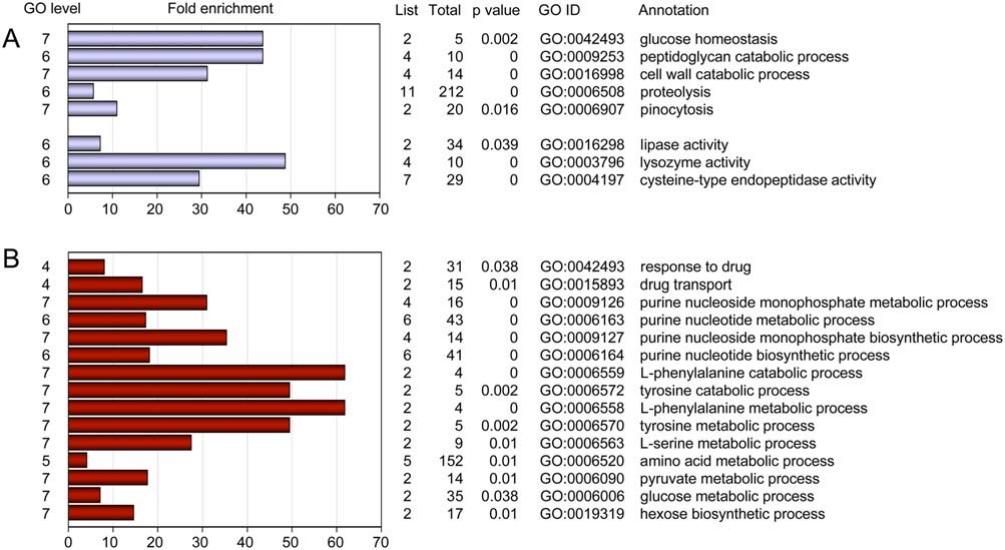
Functional annotation of CbfA-regulated genes. Shown is a selection of GO biological process and protein activity terms that were enriched in the set of 80 downregulated genes (A) and 82 upregulated genes (B) in the CbfA-deficient mutant, respectively. GO tree levels are shown on the left. GOAT calculates the target list (differentially regulated genes in a given category) and reference list (genes in a given category on the microarray) frequencies, calculates the enrichment, and the significance of the enrichment (p value). The bars indicate the fold enrichment. The table indicates the number of genes in a particular annotation (List), on the entire array (Total), the significance for enrichment (p value), the corresponding GO ID number, and the annotation.

The differential expression profile observed in the microarray experiments was confirmed for ten representative genes using real-time RT-PCR. As shown in Figure 1, differential expression of these genes was confirmed. In order to match biological functions to the list of differentially regulated genes, we performed a gene ontology (GO) term enrichment analysis using a gene ontology analysis tool (GOAT) [21], [22]. A list of GO term enrichments for genes differentially expressed in the CbfA mutant is deposited in Table S2. Our analysis suggests that CbfA positively regulates biological functions such as glucose homeostasis and the intake and digestion of food bacteria (e.g., peptidoglycan catabolic processes, cell wall catabolic processes, proteolysis, pinocytosis; Figure 2A). This is also supported by an enrichment of protein functions such as cysteine-type endopeptidase activity, lysozyme activity, or lipase activity. CbfA negatively regulates several genes involved in amino acid and purine nucleoside/nucleotide metabolism (Figure 2B). CbfA also controls the expression of two ABC transporter G family proteins involved in multidrug transport (*abcG2* and *abcG3*), and downregulates two key enzymes of gluconeogenesis, fructose-1,6-bisphosphatase (*fbpA*) and phosphoenolpyruvate carboxykinase (*pckA*).

### Functional complementation of CbfA-dependent gene expression by full-length recombinant CbfA

**Figure 3:**
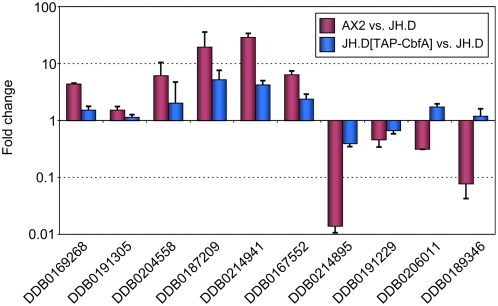
Complementation of aberrant gene expression in JH.D cells by full-length recombinant CbfA. Ten representative ESTs from the microarray data, indicated by the corresponding Dictybase (DDB) numbers, were analyzed by real-time RT-PCR. Expression of the listed genes is compared for AX2 vs. JH.D and JH.D cells expressing recombinant TAP-CbfA vs. JH.D, respectively. Note that higher expression of genes in AX2 or JH.D expressing TAP-CbfA than in JH.D results in ratios>1. The data are expressed as means of fold change±SD from three independent RNA preparations.

To further support the obtained microarray data, we performed a complementation of JH.D cells with ectopically expressed, full-length recombinant CbfA. We have previously shown that full-length CbfA expressed under the control of the constitutively active *act15* promoter was able to fully rescue the developmental defect of CbfA mutant JH.D [5]. Here, we tested whether recombinant full-length CbfA was also able to restore the aberrant expression of vegetative genes in strain JH.D. First, we cloned a cDNA encoding amino acid positions 11–1000 of CbfA into a *Dictyostelium* TAP-tag expression vector and then we performed real-time measurements of cDNA isolated from JH.D cells expressing TAP-CbfA compared with untransformed JH.D cells with the same set of ten representative CbfA-dependent genes shown in Figure 1. As shown in Figure 3, aberrant expression of the tested CbfA-dependent genes was partially complemented in the presence of recombinant full-length CbfA. Thus, we conclude that the genome-wide microarray analyses described above identified an authentic set of CbfA-dependent genes and that we can use the ten genes being tested in real-time PCR measurements as representative of the microarray study and as a useful tool to investigate how individual CbfA domains are involved in this regulation (described below).

### CbfA-CTD mediates a subset of CbfA gene regulatory functions

The first evidence in support of an autonomous gene regulatory function of the carboxy-terminal domain of CbfA (CbfA-CTD), i.e., gene regulation that does not require the remainder of the CbfA protein, came from experiments in which we constitutively expressed CbfA-CTD (CbfA^724–998^) in the background of CbfA mutant JH.D. We found that the reduced steady-state transcript levels of retrotransposon TRE5-A in the CbfA mutant were restored in the presence of CbfA^724–998^ (O.S. and T.W., manuscript in preparation).

In order to evaluate whether CbfA-CTD has a gene regulatory function beyond TRE5-A expression, we expressed a GFP fusion of CbfA-CTD (hereafter named GFP-CbfA^724–998^) in both wildtype AX2 and mutant JH.D cells, and evaluated global gene expression in both strains using DNA microarrays. Fortunately, the microarrays used in this study carry a sequence tag of the *cbfA* gene (AF052006; nucleotides 2500–3177 of the *cbfA* gene) that covers most of the CbfA-CTD-encoding region. This allowed us to compare levels of CbfA-CTD-derived mRNAs in AX2, JH.D, and transformants expressing GFP-CbfA^724–998^. We should stress here that JH.D is not a knock-out strain; instead, mutants were constructed by knock-in of a *cbfA* genomic DNA fragment containing a premature *amber* stop codon that is partially suppressed in the mutant by a co-transformed *amber* suppressor tRNA gene [8]. It was suggested from previously performed real-time RT-PCRs (unpublished data) that JH.D cells express *cbfA^am^* mRNA at a level similar to *cbfA* mRNA in wildtype cells. This observation was confirmed in this study using the microarrays, demonstrating a non-significant difference in the expression of *cbfA* sequence tag AF052006 on the microarrays when comparing AX2 and JH.D cells (data not shown). Thus, underexpession of full-length CbfA protein in JH.D cells is not due to *cbfA^am^* mRNA instability, but is rather a consequence of inefficient suppression of the premature stop codon. As a result, we determine about 5% of the full-length 115 kDa CbfA in JH.D cells on western blots, and we would expect that a truncated 52 kDa form of CbfA (CbfA^1–454^) accumulates in mutant cells. The presence of this truncated version of CbfA was in fact demonstrated by western blotting of JH.D extracts and probing with monoclonal antibody 3H7 directed against the CbfA-JmjC domain (Figure S1). It is important to note that CbfA^1–454^ is unable to enter the nuclei of JH.D cells, which we showed by western blotting of nuclear extract proteins (Figure S1). Thus, we exclude the possibility that the gene regulatory functions of CbfA-CTD described below can be due to the reconstitution of functional CbfA from CbfA^1–454^ and CbfA^724–998^.

As deduced from the microarray data, transformants AX2[GFP-CbfA^724–998^] and JH.D[GFP-CbfA^724–998^] overexpressed CbfA-CTD-containing mRNAs by a factor of 2.33 and 2.36, respectively, compared to *cbfA* mRNAs in AX2 and JH.D cells (data not shown). Similar levels of recombinant GFP-CbfA^724–998^ protein in AX2[GFP-CbfA^724–998^] and AX2[GFP-CbfA^724–998^] cells were confirmed by western blotting of whole-cell extract proteins from both transformants (data not shown).

**Figure 4:**
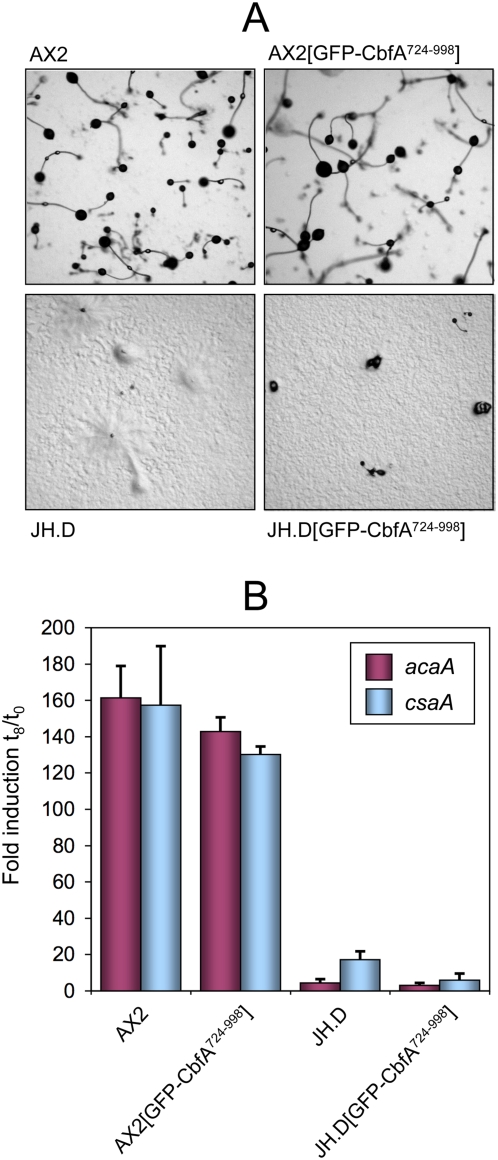
Developmental phenotype of *D. discoideum* cells expressing GFP-tagged CbfA-CTD. (A) Micrographs of the indicated *D. discoideum* strains after 30 hours of development on nutrient-free agar plates. (B) Real-time RT-PCR quantification of expression of the developmental genes *acaA* and *csaA* in the indicated strains. The values are expressed as means of induction after 8 hours of development (t_8_) versus growing cells (t_0_) in three independent RNA preparations±SD.

We have previously found that the aggregation defect of JH.D cells may be explained by the lack of mRNA expression of adenylyl cyclase ACA, such that the mutant cells fail to set up the cAMP signaling system [9]. We therefore investigated whether the expression of GFP-CbfA^724–998^ in the CbfA mutant background would be sufficient to restore ACA expression and the multicellular development of JH.D cells. This was obviously not the case; JH.D cells expressing GFP-CbfA^724–998^ retained their aggregation defect (Figure 4A) and they could neither induce their *acaA* gene nor another typical cAMP-induced aggregation marker gene, *csaA* (Figure 4B). Of note is that the expression of GFP-CbfA^724–998^ in the wildtype background of AX2 cells had no notable effect on the induction of the two cAMP-induced genes *acaA* and *csaA*, nor did it have any effect on the multicellular development of AX2 transformants (Figure 4).

In a series of DNA microarray experiments, we compared expression profiles of untransformed wildtype AX2 cells and the CbfA-deficient mutant JH.D with both strains constitutively expressing GFP-CbfA^724–998^. Applying a threshold level of 1.5 for differentially expressed genes, we noticed 79 and 88 genes as being upregulated and downregulated, respectively, in the presence of GFP-CbfA^724–998^ in JH.D cells. Although we assumed that the GFP-CbfA^724–998^ protein might exert dominant-negative effects on CbfA-mediated gene regulation, it was rather unexpected that the expression of GFP-CbfA^724–998^ in the AX2 background resulted in the differential gene expression of 235 genes, 153 of which were upregulated and 82 downregulated in the presence of CbfA-CTD. Seventy-three of these genes were not listed among the 162 CbfA-dependent genes described above, suggesting that CbfA-CTD had unspecific effects on this set of genes. Thus, we decided to omit these genes from further analyses and to focus on the 162 CbfA-dependent genes to evaluate the gene regulatory functions of CbfA-CTD.

**Figure 5:**
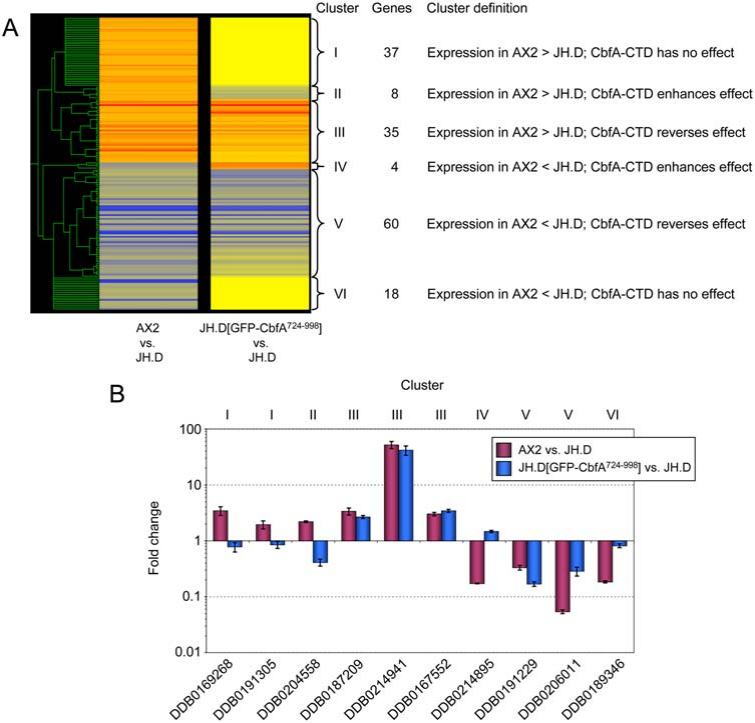
Gene regulatory effects of CbfA-CTD. (A) Left column: presentation of microarray data of 162 CbfA-regulated genes as defined by comparison of AX2 with JH.D. The colors represent the fold change (red: expression AX2>JH.D; blue: AX2<JH.D). Right column: expression of 162 CbfA-regulated genes in JH.D cells expressing GFP-CbfA^724–998^ vs. JH.D (red: expression JH.D[GFP-CbfA^724–998^]>JH.D; blue: JH.D[GFP-CbfA^724–998^]<JH.D). Non-regulated genes are displayed in yellow. The genes were clustered with GeneSpring 7.2. Six clusters of co-regulated genes were identified (cluster descriptions are given in the table to the right of the figure). The numbers of genes per cluster is indicated. (B) Verification of the microarray data using real-time RT-PCR of selected genes. We tested 2–3 representative genes for clusters that contained ≥10 genes and one gene for clusters with ≤10 genes. The corresponding cluster numbers are indicated above the figure. The data set for the AX2 vs. JH.D comparison is the same as in Figure 1. The data are expressed as means of fold change±SD from three independent RNA preparations.

The microarray data obtained from the set of 162 CbfA-regulated genes were used for a cluster analysis using the program GeneSpring 7.2 to identify co-expressed genes. The results of this analysis are shown in Figure 5A; they suggest that CbfA-CTD is capable of performing both repression and transactivation activities in the absence of the remainder of the CbfA protein. The microarray data were validated by real-time RT-PCR (Figure 5B).

The coexpression analysis suggested six different modes of CbfA-mediated gene regulation. Cluster I contains genes whose expression was downregulated in CbfA-deficient cells. These genes did not respond to CbfA-CTD expression in the mutant. Cluster II represents a small set of genes that were underexpressed in CbfA-deficient cells, and their expression was further suppressed by ectopic CbfA-CTD expression. Cluster III also represents genes that were downregulated in the absence of CbfA, but their expression was rescued by the expression of CbfA-CTD in the mutant. Cluster IV contains four genes that were overexpressed in CbfA-deficient cells, and the presence of CbfA-CTD further enhanced this overexpression phenotype. In clusters V and VI, we summarized genes that were overexpressed in CbfA-deficient cells, and the expression of CbfA-CTD resulted in either reversion or had no effect on the expression of these genes.

**Figure 6:**
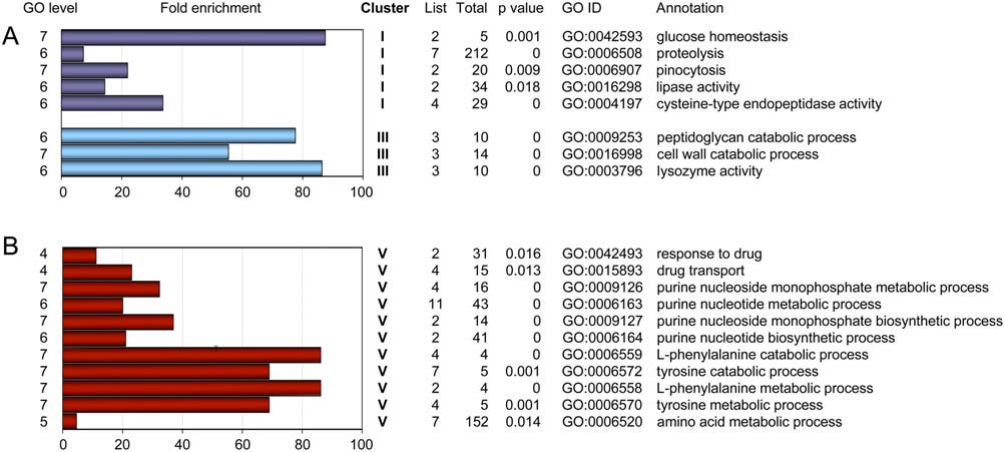
Functional annotation of coregulated, CbfA-dependent genes. Genes from clusters I–VI, as defined in Figure 5, were separately subjected to GO term enrichment analyses. Significant enrichment of GO biological process and protein activity terms was only detected for clusters I, III, and V as indicated. The bars indicate fold enrichment. The table indicates the number of genes in a particular annotation (List), on the entire array (Total), the significance for enrichment (p value), the corresponding GO ID number, and the annotation.

We perfomed GO term enrichment analyses for each individual cluster (Table S3), and found significant enrichments only in clusters I, III, and V (Figure 6). Interestingly, CbfA-CTD negatively co-regulates genes involved in nucleotide metabolism and amino acid metabolism (cluster V), whereas it positively co-regulates enzyme functions involved in bacteria degradation (cluster III).

### Functional importance of the AT-hook in CbfA-CTD

**Figure 7:**
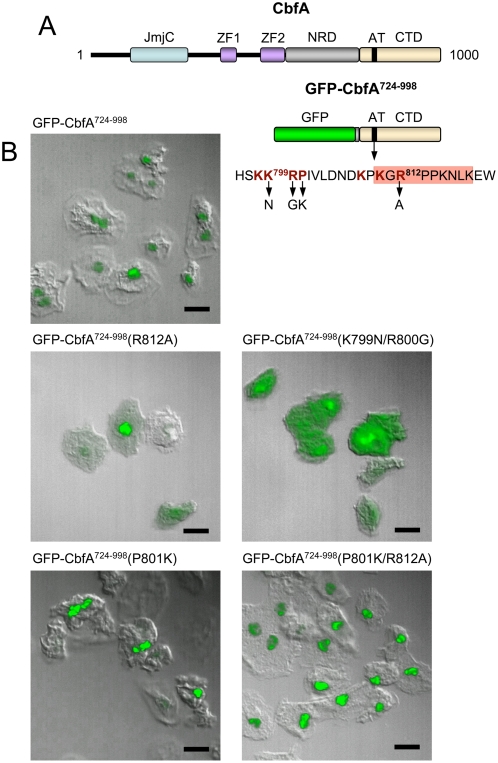
Nuclear localization of CbfA-CTD. (A) Schematic presentation of the CbfA protein. CbfA (1000 amino acids) contains the following domains: JmjC: jumonji domain; ZF: zinc fingers; NRD: asparagin-rich domain; AT: AT-hook; CTD: carboxy-terminal domain. GFP-CbfA^724–998^ represents a protein expressed from plasmid pPT132-CTD. The amino acid sequence surrounding the AT-hook is indicated. The AT-hook itself is indicated by the red box. Amino acids contributing to nuclear localization are written in red color and bold. Mutations introduced into the GFP-CbfA^724–998^ protein to test for nuclear localization and *in vivo* function of the AT-hook, respectively, are indicated below the arrows (see text for details). (B) Shown is the expression in JH.D cells of GFP-CbfA^724–998^ wildtype protein and mutants GFP-CbfA^724–998^(R812A), GFP-CbfA^724–998^(K799N/R800G), GFP-CbfA^724–998^(P801K), and GFP-CbfA^724–998^(P801K/R812A). Cells were fixed and analyzed by confocal laser scanning microscopy. Scale bar is 10 µm.

We have previously shown that the AT-hook is required for the specific binding of bacterially expressed CbfA-CTD protein to DNA *in vitro* [7]. Thus, it was tempting to evaluate whether the gene regulatory effects of CbfA-CTD on cluster III and V genes were dependent on a functional AT-hook *in vivo*. At this point it was important to consider that if CbfA-CTD had autonomous gene regulatory functions, as described above, then the protein must be expected to enter the nucleus without the help of the remainder of the CbfA protein. Therefore, the compartmentalization of the GFP-tagged CbfA-CTD (Figure 7A) in *D. discoideum* cells was analyzed by fluorescence microscopy. We observed that the GFP-CbfA^724–998^ fusion protein quantitatively accumulated in the nuclei of *D. discoideum* cells (Figure 7B). Nuclear enrichment of a non-tagged version of the CbfA^724–998^ protein was independently confirmed by preparation of nuclear extracts from JH.D transformants (see Figure S1).

Interestingly, *in silico* analysis predict two nuclear localization sequences (NLS) within the CbfA protein, one of which overlaps with the AT-hook motif. The first putative NLS (SSQ**K^488^K**IKCHRCE**KR**F**KK**FS; NLS written in bold) is located outside of the CbfA-CTD as part of zinc finger region 2. The second putative NLS is located within the CbfA-CTD and covers the AT-hook within the sequence **KK^799^RP**IVLDND**K**P**K** G**R^812^**PPKNLKEW (putative NLS in bold; AT-hook underlined).

**Figure 8:**
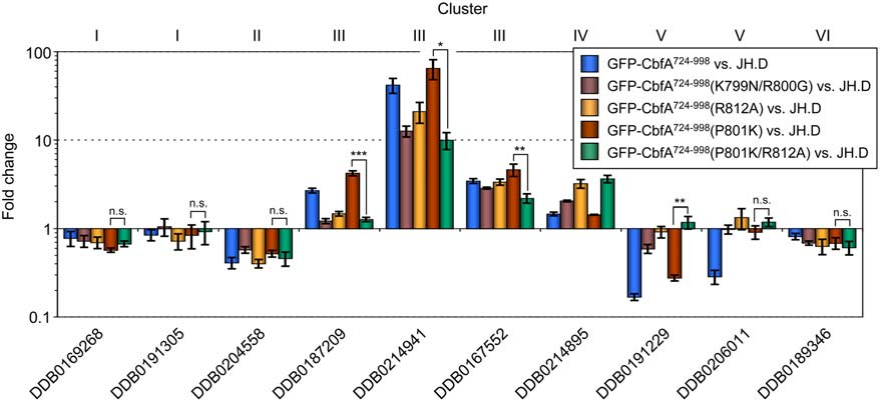
Impact of the AT-hook on gene regulatory activity of CbfA-CTD. Real-time RT-PCR of ten representative CbfA-dependent genes. The corresponding cluster numbers are defined in Figure 5. The data are expressed as means of fold change±SD from three independent RNA preparations. * p<0.05; ** p<0.005; *** p<0.0005; n.s., not significant (p>0.05) (two-tailed t-test).

The bipartite NLS-2 was analyzed in some detail by site-directed mutagenesis. First, we introduced into the GFP-CbfA^724–998^ protein the R812A mutation that was previously shown to compromise the DNA binding capacity of CbfA-CTD [7]. The GFP-CbfA^724–998^(R812A) protein was then expressed in CbfA-depleted JH.D cells. As shown in Figure 7B, the protein was still enriched in the nucleus, but a significantly higher amount of protein was cytoplasmic as compared to the wildtype CbfA-CTD protein. This suggested that R812 may in fact contribute to the bipartite NLS-2. Although high levels of the GFP-CbfA^724–998^(R812A) protein appeared in the nucleus, the mutant protein regulated the expression of cluster III and V genes with significantly lower capacity than the wildtype CbfA-CTD protein (Figure 8). When lysine-799 and arginine-800 (K^799^R^800^) of the bipartite NLS-2 were changed to asparagine and glycine, respectively, the resulting GFP-CbfA^724–998^(K799N/R800G) mutant protein was present in the cytoplasm but also accumulated in the nuclei (Figure 7B). As a consequence, the efficacy of this mutant protein to mediate gene regulation was reduced (Figure 8). In summary, the postulated NLS-2 is functional, but neither of the tested mutations alone could completely abolish nuclear enrichment of the CbfA-CTD protein.

To confirm the importance of an intact AT-hook for gene regulatory functions of CbfA-CTD *in vivo*, we wanted to further enrich an AT-hook mutant protein (R812A) in the nucleus. First, we changed the KKRP^801^ sequence of NLS-2 into KKRK^801^ in the GFP-CbfA^724–998^ protein, leaving the AT-hook intact. As expected, this mutant protein accumulated quantitatively in the nucleus (Figure 7B) and was able to fully restore CbfA-CTD-dependent gene regulation (Figure 8). Next, we generated a P801K/R812A double mutant. This protein quantitatively entered the nucleus (Figure 7B), but, as shown in Figure 8, expression of CbfA-CTD-dependent genes was reduced by 52–85% compared to the KKRK^801^ mutant in the tested cluster III genes. There seems to be a similar response by cluster V genes, although gene DDB0206011 did not respond to CbfA-CTD expression in this particular experiment (Figure 8; compare Figure 5B). These data strongly suggest that CbfA-CTD exerts its gene regulatory functions *in vivo* by binding to DNA via the AT-hook.

## Discussion

In this study we identified two types of CbfA-dependent genes. The first group of genes, combined in clusters I, II, IV, and VI (Figure 5), probably depends on the activity of full-length CbfA, at least including its JmjC/ZF domains. Although we cannot discriminate yet whether regulation of these genes also requires the CbfA-CTD, we assume that this activity of CbfA involves chromatin remodeling catalyzed by the CbfA-JmjC domain. The second set of CbfA-dependent genes (clusters III and V) requires only the CbfA-CTD for proper expression, and is likely to be regulated without the requirement of CbfA-mediated chromatin modifications. Cluster III genes, which are positively regulated by CbfA-CTD, are enriched in GO terms representing functions that support bacterial food uptake and degradation, such as peptidoglycan degradation (e.g., lysozyme activity) and proteolysis. On the other hand, CbfA-CTD downregulates cluster V genes, which encode functions involved in purine nucleotide and amino acid metabolism, as well as drug transport.

The obtained data suggest that CbfA is an important housekeeping transcription regulator in *D. discoideum* cells. That is, it supports the expression of gene products required in life phases where food is plenty and energy and metabolites can be acquired by food intake rather than by the breakdown of intracellular metabolites. CbfA-depleted cells hardly grow on bacteria due to a phagocytosis defect [8], suggesting that CbfA may regulate the expression of genes important in phagocytosis. Sillo et al. [23] have recently reported a detailed analysis of *D. discoideum* genes involved in the regulation of phagocytosis of bacteria by *D. discoideum* amoebae. Analyzing this data set, we found that among the list of 443 genes reported by Sillo et al. [23] to be responsive to a switch from axenic growth of *D. discoideum* cells to cultivation with bacteria, five genes were identified in our study to be expressed in a CbfA-dependent manner. Among those, four genes were downregulated in CbfA-depleted cells (DDB0167552, DDB0169107, DDB0187209, DDB0215459). One of these genes (DDB0167552) encodes a glycoside hydrolase family 25 protein, a putative extracellular protein with lysozyme activity. DDB0169107 encodes the S17 protein of the small ribosomal subunit, while DDB0187209 encodes a protein of unknown function with a frizzled domain. One gene of unknown function (DDB0219898), which is 11-fold upregulated by the incubation of *D. discoideum* cells with bacteria [23], is about 2-fold upregulated in CbfA-depleted cells.

Our data suggest that the AT-hook of CbfA-CTD is required to exert its gene-regulatory functions. In the experiment presented in Figure 8, substitution of arginine-812 within the GRP motif of the AT-hook had a significant effect on the gene regulatory capacity of CbfA-CTD, although its activity was not completely abolished. It has been noted that the arginine residues in the AT-hook sequence P**R**G**R**P insert their side chains into the minor groove of DNA and thus contribute directly to DNA binding, while lysines flanking the AT-hook core contribute to DNA binding by neutralizing negative charges of the DNA backbone [13], [17]. Interestingly, the AT-hook in the CbfA protein has the rather unusual core motif P**K**G**R**P, Residual activity of the R812A mutant of CbfA-CTD may be explained if we assume that at least lysine-810 within the AT-hook sequence **K**P**K^810^**G**R**PP**K**NL**K** of CbfA directly contributes to the specificity of DNA binding of CbfA-CTD *in vivo*.

It is worth noting that CbfA-CTD, if expressed in AX2 cells, had no significant effect in the microarray and real-time RT-PCR experiments on any of the genes represented in clusters I–VI. Although this may be expected for genes of clusters I, II, IV, and VI, which are expressed in a CbfA-dependent but perhaps CbfA-CTD-independent manner, it was surprising to find that CbfA-CTD also had no effects on cluster III and V genes in AX2 cells, which strongly responded to CbfA-CTD expression in JH.D cells (as shown in Figure 5). There may be two explanations for this observation. On the one hand, regulation of cluster III and V genes by CbfA may not involve any direct binding to the respective gene promoters, but instead require protein interactions in multiprotein transcription complexes that cannot be accessed by isolated CbfA-CTD. Alternatively, isolated CbfA-CTD may have a reduced affinity for its target promoter sequences and may be unable to displace endogenous CbfA. Since we have found that the CbfA-CTD-mediated effects on the expression of cluster III and V genes requires the AT-hook, we can hypothesize that in wildtype cells, CbfA-CTD competes with endogenous CbfA for binding sites on genomic DNA, but that binding of either protein results in normal gene activity, such that no differential expression is observed. This hypothesis would also imply that CbfA-dependent regulation of gene clusters I, II, IV, and VI may occur without direct binding of CbfA to the respective promoters.

In DNA footprinting analyses using the C-module of retrotransposon TRE5-A as probe, two CbfA binding sites have been determined that contain 14-22 homopolymeric thymidines [6]. Given that the *D. discoideum* genome contains 78% A+T and a plethora of oligothymidine motifs [19], it remains an open question how CbfA manages to exactly recognize its specific DNA targets *in vivo*, while ignoring others. High concentrations of minor groove-binding drugs such as distamycin, which are known to alter DNA structure at high concentrations [24], abolished CbfA binding to the C-module *in vitro* [7]. In addition, while the CbfA binding site mapped in the *acaA* upstream region contains an oligo(dT) tract of 52 nucleotides length, its interruption by blocks of 10 adenine bases abolished the binding of CbfA to the altered DNA fragment in gel shift assays [9]. On the other hand, CbfA binding to either the C-module or the *acaA* upstream region *in vitro* was unaffected by a large excess of *D. discoideum* promoter sequences that contain multiple oligo(dT) stretches of variable lengths. In summary, it seems that the local DNA structure, given by the length of oligo(dT) and the surrounding sequences, defines a CbfA binding site *in vitro* (i.e., on naked DNA) and maybe also *in vivo*. The results shown here suggest that the *in vitro* DNA binding specificity of CbfA translates to the *in vivo* situation in that CbfA binding to target sequences occurs at the AT-hook of the CbfA-CTD, is DNA sequence-specific, and apparently occurs only a very small and distinct number of genomic loci.

The results obtained in this study may provide some insight to the adaptations of transcription factors to rapidly evolving genomes. A high plasticity of transcription factor domain structures may be required, on the one hand, to maintain existing gene regulatory networks. This plasticity of transcription factors may be, on the other hand, a source of new gene regulatory functions generated by either rapid evolution of existing domains and/or domain swapping within existing transcription factors. In the context of the data presented in this study, we hypothesize that CbfA arose in ancient dictyostelids from a common ancestor in which repeated CTD swapping and/or rapid evolution of CTDs, while conservating the JmjC/ZF architecture, generated new gene regulatory functions of CbfA-like proteins, i.e. CbfA and CbfB. Support for this assumption comes from the following considerations. First, *D. discoideum* CbfA and CbfB are paralogous within the JmjC/ZF region, but have completely unrelated carboxy-terminal domains. Second, database searches using full-length *D. discoideum* CbfA identified several “CbfA-like proteins” in filamentous fungi (but not in yeasts, animals, or plants) that share the exact JmjC domain and zinc finger topology of CbfA at about 25% amino acid identity level (O.S. and T.W., manuscript in preparation). However, these CbfA-like fungal proteins have completely diverged carboxy-terminal domains when compared to each other and to the CbfA or CbfB proteins from *D. discoideum*. Thus, the domain structures of “CbfA-like” proteins are dynamic in evolution and may be a source for the altered regulation of gene networks that drive the evolution of cell functions, at least in dictyostelids and fungi.

## Methods

### CbfA-specific antibodies

Generation of monoclonal antibody 7F3, raised against a bacterially expressed protein covering amino acids 795–998 of CbfA (CbfA-CTD), was described elsewhere [8]. Antibody 3H7 was generated by immunizing mice with a bacterially expressed protein covering amino acids 78–355 of CbfA as previously described for 7F3 [8]. Animals were housed in special animal facilities at the Biocenter of the University of Frankfurt under standard conditions. Mice mice were maintained on a 12-h dark–light cycle with pelleted food and tap water ad libitum. All experiments were carried out according to the European Communities Council Directive (86/609/EEC) by individuals with appropriate training and experience.

### *Dictyostelium* cell culture and transformants

In most experiments we used a GFP-tagged CbfA-CTD, which covered amino acids 724–998 of CbfA. Note that the former description of amino acid positions in CbfA [8] was misleading due to an unnoticed fusion of two genomic DNA fragments in the genomic library used to isolate *cbfA* sequences. The corrected protein sequence of CbfA, which covers 1000 amino acids, can be found in Genbank under accession number AF052006. The expression vector used to express GFP-CbfA^724–998^, pPT132-CTD, was constructed by Gateway recombination of a PCR fragment (primers attB1-CMBF01 and attB2-CMBF-02; see Table S4) corresponding to amino acid positions 724–998 of CbfA into vector pPT132 [25]. Amino acid substitutions to characterize a potential nuclear localization sequence and the AT-hook of CbfA-CTD were obtained by site-directed mutagenesis of plasmid pPT132-CTD. In some experiments we used an untagged version of the GFP-CbfA^724–998^ protein. Vector pDXA-CTD was generated by cloning a PCR fragment encoding CbfA^724–998^ (primers CMBF-37.3 and CMBF40.1, see Table S4) into the KpnI and XhoI sites of pDXA-3H [26]. For complementation analyses we also expressed a nearly full-length CbfA protein in *D. discoideum* cells. This protein was tagged at its amino terminus with a tandem affinity purification tag (TAP) and yellow fluorescent protein (YFP) derived from vector pDV-NTAP-NYFP [27]. First, a genomic PCR fragment spanning nucleotides 182–3004 of the *cbfA* gene (corresponding to exon 2) and 699 nucleotides of downstream region was amplified by PCR using primers cAK-01 and cAK-02 and cloned into pGEM-T (pGEM-cbfA01). Next, 520 bp of *cbfA* upstream region were amplified with primers cAK-03 and cAK-04 and inserted as NcoI/BglII fragment into pGEM-cbfA01 (resulting in pGEM-cbfA02). Finally, an NTAP/NYFP fragment was amplified from vector pDV-NTAP-NYFP as BamHI fragment (primers cAK-05 and cAK-06) and ligated into the BglII site of pGEM-cbfA02. The fusion protein derived from vector pGEM-cbfA03 spans amino acids 11–1000 of CbfA and is named TAP-CbfA.

*D. discoideum* AX2 (wildtype cells ) and *cbfA^am^* mutant JH.D [8] were grown in HL5 medium as described [8]. Expression vectors were transformed into AX2 and JH.D and G418-resistent clones were recovered as described previously [8].

### Western blots

Expression of CbfA was analyzed as described previously [8], [28]. Briefly, logarithmically growing *D. discoideum* cells were washed twice in 17 mM phosphate buffer (pH 6.2) and kept as frozen pellets of 2×10^7^ cells at −80°C. About 50 µg per lane of whole-cell extract proteins were separated by SDS-PAGE and CbfA was stained using either the CbfA-CTD-specific monoclonal antibody 7F3 or the CbfA-JmjC-specific antibody 3H7. Nuclear extracts were prepared as described in Siol et al. [9].

### Fluorescence microscopy

Cellular localization of the GFP-CbfA^724–998^ proteins was analyzed by seeding *D. discoideum* cells to 20% confluence in petri dishes containing sterile coverslips. After 24 hours of cultivation, the cells were fixed by replacing HL5 medium with 3.8% paraformaldehyde in PBS for 30 minutes. Then, the coverslips were mounted with AntiFade and placed onto microscope slides and GFP fluorescence was observed under an LSM 5 live instrument (Carl Zeiss).

### DNA microarrays

A complete description of the microarray dataset, as described by Na et al. [18], is available at the Gene Expression Omnibus (GEO; accession number GPL1972). The microarray experiments were performed and data were analyzed essentially as described previously [18], [20]. For each set of experiments, we used three independent RNA preparations, hybridized six microarrays, and swapped the dyes in the labeling reaction to exclude dye-specific errors. GO term enrichment analyses were performed as described [22].

### Real-time RT-PCR

For a complete list of real-time RT-PCR primers used in this study, see Table S4. Logarithmically growing *D. discoideum* cells were washed in 17 mM phosphate buffer (pH 6.2) and stored as pellets of 2×10^7^ cells at −80°C until further use. Total RNA was prepared from frozen cells using the Qiagen RNeasy® Mini kit according to the provided protocol. cDNA was synthesized by reverse transcription of 500 ng of total RNA using an oligodesoxythymidine primer and the Qiagen Omniscript® RT kit. The *acaA* gene was amplified from cDNA as a 172 bp fragment with primers acaA-09 and acaA-04. A 182 bp fragment of the *csaA* gene was amplified from cDNA using primers csaA-05 and csaA-06. Real-time PCR signals were standardized for expression of the gene encoding GAPDH (*gpdA*, Dictybase entry DDB0185087; [29]). The *gpdA* gene was amplified with primers gpdA-01 and gpdA-02, yielding a 247 bp PCR product from genomic DNA and a 156 bp fragment from cDNA, respectively. Thus, amplification of *gpdA* was also suitable to determine genomic DNA contaminations in cDNA preparations by conventional RT-PCR prior to real-time RT-PCR runs. Real-time amplification was carried out using the Stratagene Brilliant® SYBR® Green QPCR Master Mix on a Stratagene Mx3000P instrument. After an initial denaturing step at 95°C for 10 minutes, the PCR was performed for 40 cycles at 95°C for 30 s, 58°C for 30 s and 72°C for 30 s. Regulation was calculated with the method of Pfaffl [30] using GAPDH as a reference gene.

## Supporting Information

Figure S1 Cellular compartmentalization of CbfA, the premature translation product of CbfA, and CbfA-CTD.
(0.43 MB PDF)

Table S1 List of CbfA-regulated genes. Differential gene expression in AX2 vs. JH.D and response to CbfA-CTD expressed in JH.D cells.
(0.04 MB XLS)

Table S2 List of enriched biological process, molecular function and cellular component GO terms between AX2 and JH.D.
(0.05 MB XLS)

Table S3 List of enriched biological process, molecular function, and cellular component GO terms in comparison of AX2 vs. JH.D and JH.D[CbfA-CTD] vs. JH.D.
(0.05 MB XLS)

Table S4 List of primers used in this study.
(0.04 MB PDF)
