# Supplementary material for: The Carboxy-Terminal Domain of Dictyostelium C-Module-Binding Factor Is an Independent Gene Regulatory Entity
Source: PLoS One. 2009 Apr 3;4(4):e5012. doi: 10.1371/journal.pone.0005012 (PMC2661138; doi:10.1371/journal.pone.0005012)
Supplement: Figure S1 — Cellular compartmentalization of CbfA, the premature translation product of CbfA, and CbfA-CTD. (0.43 MB PDF) [file pone.0005012.s001.pdf]

Supplemental Figure 1: Cellular compartmentalization of CbfA, the premature translation product of CbfA, and CbfA$^{724\text{-}998}$.

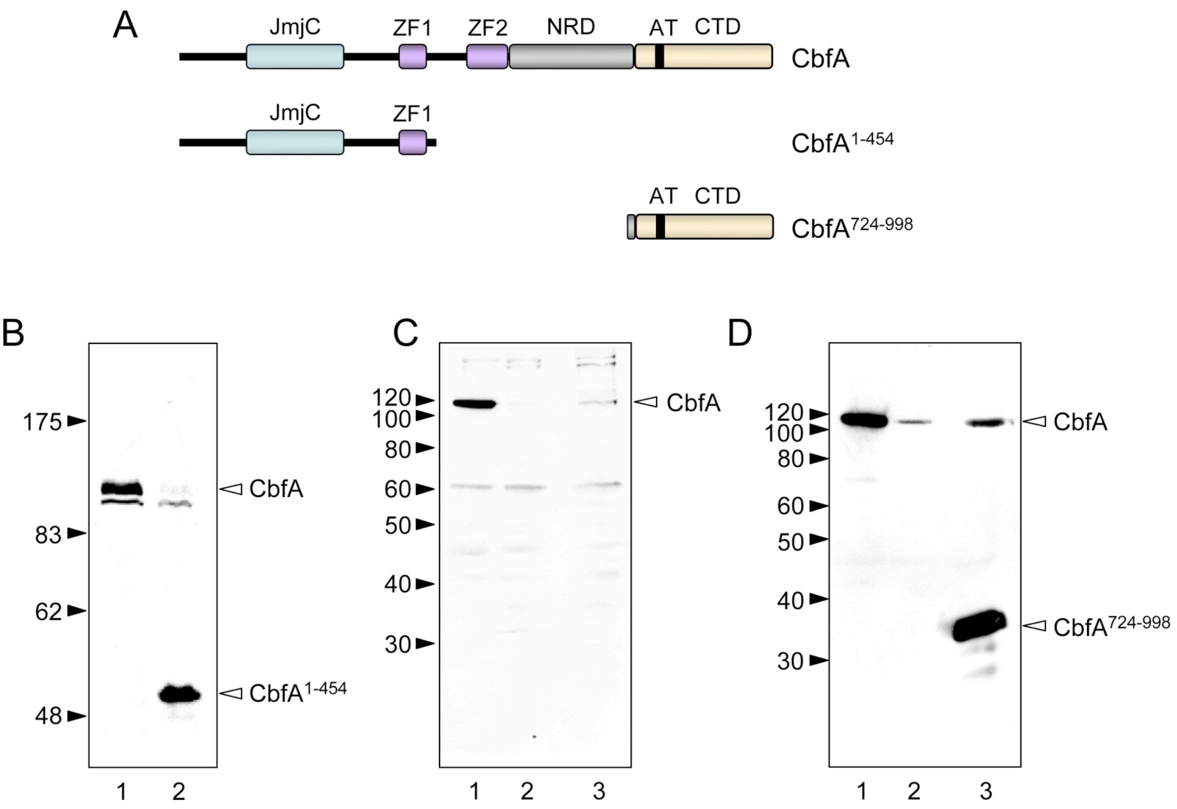

(A) Schematic presentation of the CbfA protein. The domains are indicated (JmjC: jumonji domain; ZF: zinc finger domains; NRD: asparagine-rich domain; AT: AT-hook; CTD: carboxy-terminal domain). The CbfA-depleted mutant JH.D was generated as a knock-in of a *cbfA* gene fragment that introduced a premature *amber* translation stop codon at positions 1766-1768 (termed *cbfA^{am}* gene). The *amber* codon is partially suppressed by an UAG suppressor tRNA gene present in the same cell [1]. Since suppression occurs at low rate, the cells produce about 95 % of a truncated CbfA protein (CbfA$^{1\text{-}452}$, 52 kDa) and 5 % of full-length CbfA (115 kDa).
(B) Western blot of CbfA in whole-cell extract of AX2 (lane 1) and JH.D (lane 2) cells. CbfA was stained with monoclonal antibody 3H7, which was raised against the JmjC domain. See main text for methods.
(C, D) Enrichment of CbfA, CbfA$^{1\text{-}454}$, and CbfA$^{724\text{-}998}$ in nuclear extracts. Nuclear extracts were prepared from AX2 (lane 1), JH.D (lane 2), and JH.D[CbfA$^{724\text{-}998}$] cells as decribed [1]. JH.D[CbfA$^{724\text{-}998}$] was expressed from plasmid pDXA-CTD. CbfA was stained either with antibody 3H7 (C) or 7F3 (D). See main text for experimental details.

[1] Winckler T, Trautwein C, Tschepke C, Neuhäuser C, Zündorf I, et al. (2001) Gene function analysis by *amber* stop codon suppression: CMBF is a nuclear protein that supports growth and development of *Dictyostelium* amoebae. J Mol Biol: 305: 703-714.
