# Supplementary material for: The Carboxy-Terminal Domain of Dictyostelium C-Module-Binding Factor Is an Independent Gene Regulatory Entity
Source: PLoS One. 2009 Apr 3;4(4):e5012. doi: 10.1371/journal.pone.0005012 (PMC2661138; doi:10.1371/journal.pone.0005012)
Supplement: Table S4 — List of primers used in this study. (0.04 MB PDF) [file pone.0005012.s005.pdf]

Supplemental Table 4: List of primers used in this study.

Primer	Sequence 5'→3'
acaA-04	CATTCTAGAGGCGGTATTGGCAGTATCACC
acaA-09	GGAGAAAATGTCTGATTTCGCTTTGGATG
csaA-05	GAAAGCTGGTATCTCAAATGTTGTCAC
csaA-06	GGAATCTGGAGCACAAACTATATCAGTAG
gpdA-01	GGTTGTCCCAATTGGTATTAATGG
gpdA-02	CCGTGGGTTGAATCATATTTGAAC
DDB0169268-01	CACCTCGTGTTGGTGATTCTGT
DDB0169268-02	CGCATGTTTGGTCTTCACCACT
DDB0191305-01	CCACAACATTTGACAACTCAAAC
DDB0191305-02	GCTGGTG CAGTTTCTTTTGG
DDB0204558-01	TCAACAGTTAGGTGGTGGCTGT
DDB0204558-02	GGCGGGACTGTTACAAGCAAAT
DDB0187209-01	TGGTTGTAATTGTGCACCAGCC
DDB0187209-02	TTGGGCAAAGGTTAGCACATGG
DDB0214941-01	CACACATGGAAACAAACTCTCACTG
DDB0214941-02	CACGATCTATTTGTTGGATATATCTC
DDB0167552-01	GGATGTCACAATCAGAAAATGTTGC
DDB0167552-02	CTACCACCAGTGTATCCACC
DDB0214895-01	CACTTGGTCAAGTTGTATCTGC
DDB0214895-02	CGG CATGGATTTGAATGATAAACG
DDB0191229-01	CCATTCTTTTACCACTCCTTCATCTC
DDB0191229-02	TAGCGATTGAAGCTGGTGG
DDB0206011-01	ACATTTAGCATCCTCACCACC
DDB0206011-02	GTGAGCAATACCATAGAGATGATG
DDB0189346-01	TGATGGTACTCGTGTTCCAG
DDB0189346-02	GAGAGTTGAAGAGATACGCC
CMBF-37.3	GGGGTACCGGTTTAATGTCTCATTTTTCTGG
CMBF-40.1	CCCTCGAGTTATTTTTTATTATTATAAAAGTTCTTGTTTTAC
attB1-CMBF01	GGGGACAAGTTTGTACAAAAAAGCAGGCTTCACCATGGGTTTAATGTCTCATTTTTCTGG
attB2-CMBF02	GGGGACCACTTTGTACAAGAAAGCTGGGTTATTTTTTATTATTATAAAGTTCTTGTTTTACATATAAACC
cAK-01	AGATCTACCGGTGGATCCGAAAGATATTCAGAAATGGAATTAATTAATTCACC

cAK-02	ACCGGTAATCAAGTTGGAAATTTGAAAAGAATACTCAAG
cAK-03	CCATGGCAAATTATTTTGTACCGTGTATATACAATAATTG
cAK-04	AGATCTCATATTAAAATTAAGTATATTATCAACACTAGG
cAK-05	GGATCCTCTAGAGCAGGCCTTGCGCAACACGATG
cAK-06	CGGATCCAGTCTTGTACAGCTCGTCCATGC
